# Isotonic ion replacement can lower the threshold for selective infrared neural inhibition

**DOI:** 10.1117/1.NPh.8.1.015005

**Published:** 2021-02-19

**Authors:** Junqi Zhuo, Zihui Ou, Yuhan Zhang, Elizabeth M. Jackson, Sachin S. Shankar, Matthew T. McPheeters, Jeremy B. Ford, E. Duco Jansen, Hillel J. Chiel, Michael W. Jenkins

**Affiliations:** a Case Western Reserve University, Department of Biomedical Engineering, Cleveland, Ohio, United States; b Case Western Reserve University, Department of Biology, Cleveland, Ohio, United States; c Vanderbilt University, Department of Biomedical Engineering, Nashville, Tennessee, United States; d Vanderbilt University, Biophotonics Center, Nashville, Tennessee, United States; e Vanderbilt University, Department of Neurological Surgery, Nashville, Tennessee, United States; f Case Western Reserve University, Department of Neurosciences, Cleveland, Ohio, United States; g Case Western Reserve University, Department of Pediatrics, Cleveland, Ohio, United States

**Keywords:** infrared neural inhibition, glucose block, size selectivity, infrared neuromodulation, neurophotonics

## Abstract

**Significance:** Infrared (IR) inhibition can selectively block peripheral sensory nerve fibers, a potential treatment for autonomic-dysfunction-related diseases (e.g., neuropathic pain and interstitial cystitis). Lowering the IR inhibition threshold can increase its translational potentials.

**Aim:** Infrared induces inhibition by enhancing potassium channel activation. We hypothesized that the IR dose threshold could be reduced by combining it with isotonic ion replacement.

**Approach:** We tested the IR inhibition threshold on the pleural-abdominal connective of *Aplysia californica*. Using a customized chamber system, the IR inhibition was applied either in normal saline or in isotonic ion-replaced saline, which could be high glucose saline, high choline saline, or high glucose/high choline saline. Each modified saline was at a subthreshold concentration for inhibiting neural conduction.

**Results:** We showed that isotonically replacing ions in saline with glucose and/or choline can reduce the IR threshold and temperature threshold of neural inhibition. Furthermore, the size selectivity of IR inhibition was preserved when combined with high glucose/high choline saline.

**Conclusions:** The present work of IR inhibition combined with isotonic ion replacement will guide further development of a more effective size-selective IR inhibition modality for future research and translational applications.

## 1 Introduction

Inhibition of peripheral nerves can be useful for treating disease (e.g., pain,^1^ persistent hypertension,^2^ or obesity^3^). We showed that infrared (IR) light can block action potential propagation in both neural and cardiac tissues ^4^^–^^7^ and others have confirmed these findings.^8^^–^^12^ Unlike IR stimulation, which depends on spatiotemporal thermal gradients (dT/dt,dT/dz),^13^^–^^17^ studies suggest that IR inhibition is due to an IR-induced baseline temperature increase.^18^ Recently, we showed that temperature increases lead to rate increases in Hodgkin–Huxley gating mechanisms so that the $K^{+}$ channel activation rate overwhelms the ${\mathrm{Na}}^{+}$ channel activation rate.^19^^,^^20^ We have modeled, experimentally demonstrated, and mathematically proven that IR inhibition can preferentially block action potential propagation in small-diameter sensory fibers.^5^^,^^19^^,^^20^

We have explored ways to apply IR inhibition effectively and safely for translational applications.^21^ We showed that IR inhibition is safe and reversible in previous acute studies.^4^^,^^5^ In addition to optimizing the IR inhibition protocol (e.g., changing the block length^21^), we are exploring whether other inhibition modalities could be combined to reduce the IR threshold. For example, combining IR with electrical current lowered the threshold for IR neural stimulation.^6^^,^^15^ Similarly, we hypothesized that adding another inhibitory modality could reduce the IR inhibition threshold, enhancing potential translational applications.

Since IR inhibition speeds up the gating of voltage-gated potassium channels,^19^^,^^20^ adding a different modality that works via the axon membrane, such as affecting the voltage-gated sodium channels, could demonstrate synergy. Glucose block, which replaces isotonic glucose solution for normal saline, has been used for safe and reversible neural conduction inhibition since the 1930s.^22^^–^^25^ Similarly, choline chloride has been used for sodium-ion substitution. Using the pleural-abdominal connective of *Aplysia californica*, we tested the hypothesis that replacing part of the saline with isotonic glucose or choline solution can reduce currents through ion-selective channels and lower the IR inhibition threshold. An additional test with both glucose and choline was conducted to explore if there is any synergetic effect on the IR threshold reduction when both isotonic ion substitutions were used.

## 2 Method

### 2.1 Animal Preparation

We used the pleural-abdominal connectives from *Aplysia californica* (298±58  g, South Coast Bio-Marine, California), a nerve that consists solely of unmyelinated axons. The nerve on both sides was dissected out after anesthetizing the animal with magnesium chloride solution (333 mM, 50% body weight). Nerves were kept in normal *Aplysia* saline (460 mM NaCl, 10 mM KCl, 10 mM MOPS, 10 mM glucose, 22 mM ${\mathrm{MgCl}}_{2}{\cdot 6H}_{2}O$, 33 mM ${\mathrm{MgSO}}_{4}{\cdot 7H}_{2}O$, 13 mM ${\mathrm{CaCl}}_{2}$, pH 7.5) at room temperature (∼22°C).

### 2.2 Experimental Setup

#### 2.2.1 Electrophysiology

**Fig. 1:**
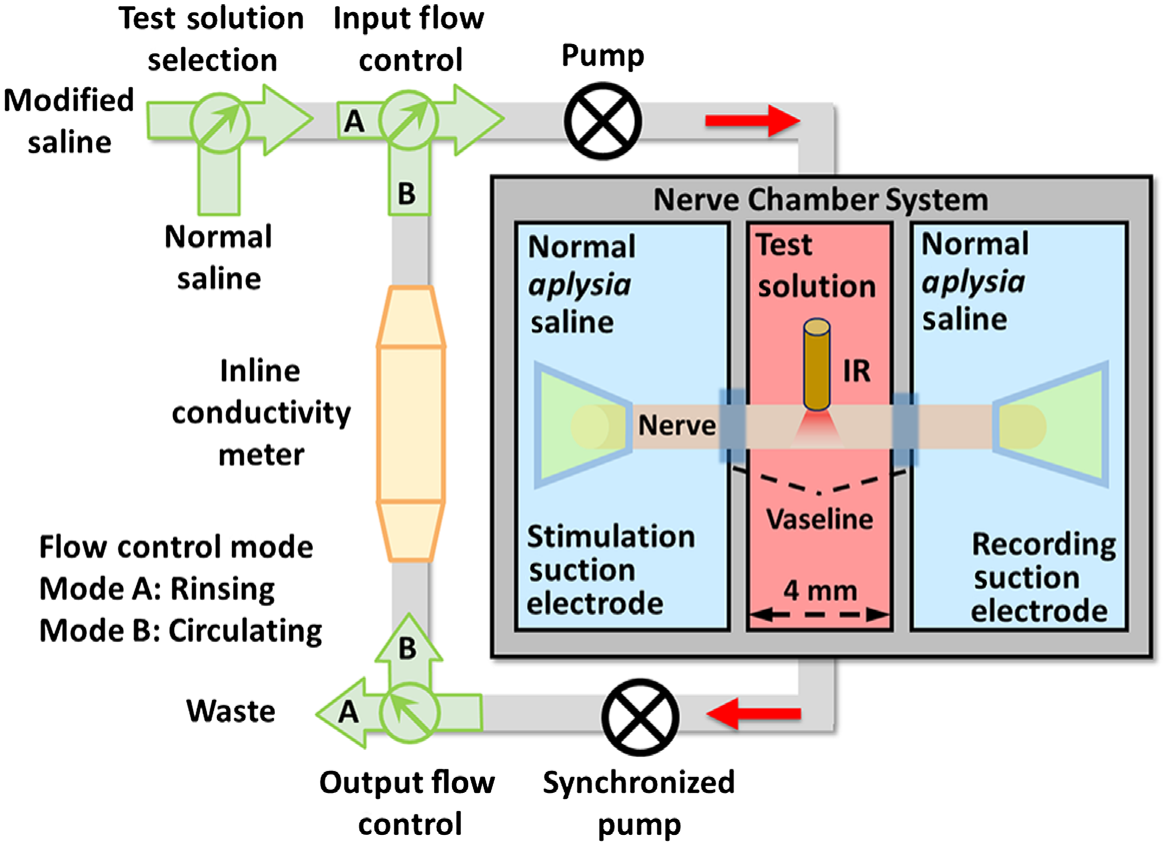
The experimental setup. The system shown schematically makes it possible to position a nerve across three isolated chambers. In the two outer chambers, the nerve was stimulated and recorded using suction electrodes in normal *Aplysia* saline. In the middle chamber, a 600-μm optical fiber in contact with the nerve delivered IR light at the same time that nerve could be bathed in either normal saline or modified saline. The modified saline could be high-glucose saline, high-choline saline, or high-glucose/high-choline saline, each of which was at a subthreshold concentration for inhibiting neural conduction. Perfusion of either the modified saline or normal *Aplysia* saline was monitored by an inline conductivity meter.

Electrical stimulation and recording via suction electrodes (0.35-mm inner diameter) were used to assess neural conduction (Fig. 1). The nerve was stimulated with bipolar electrical current pulses (1 Hz, 2 ms, 0.1 to 0.5 mA) generated by a pulse stimulator (Model 2100, A-M Systems, Washington) and delivered by a stimulus isolator (A395, World Precision Instruments, Florida). The current was adjusted to ensure that a full compound action potential (CAP) was evoked. The evoked CAPs were amplified and band-pass filtered (×10,000, 100 to 500 Hz) by a differential ac amplifier (Model 1700, A-M Systems, Washington). The amplified signal was recorded by a data acquisition (DAQ) device (USB-6003, National Instruments, Texas) using the AxoGraph X software (AxoGraph, California).

#### 2.2.2 Modified saline and fluid control

In the chamber system, the test nerve was placed in a groove that went across three chambers, while the middle chamber was perfused with different solutions using a peristaltic pump (7524-10, Barnant Co, Illinois), allowing the simultaneous application of both inhibitory modalities (Fig. 1). The solution was either normal *Aplysia* saline (in which the standard glucose concentration is 10  mM/l) or one of the modified saline solutions:

1. High glucose saline: 20% v/v of isotonic glucose solution mixed with 80% v/v normal *Aplysia* saline, in which all the ion concentrations were diluted to 80% of the level in normal *Aplysia* saline. The elevated glucose concentration (235  mM/l) was the minimum level that did not cause significant inhibition or spontaneous activity in our preliminary test. In the same test, the threshold for acute inhibition with glucose was 1105±20  mM/l (N=5, data not shown).
2. High choline saline: 20% v/v of isotonic choline chloride solution mixed with 80% v/v normal saline. The choline chloride concentration (113  mM/l) was selected to match the osmotic concentration of the additional glucose in the high glucose saline, so the dilution to the other ions was the same (80%). Since we are using choline chloride, the chloride concentration was maintained instead of being diluted, which does not have a significant effect on neural conduction.
3. High glucose/high choline saline: 10% v/v of isotonic glucose solution and 10% v/v of isotonic choline chloride solution mixed with 80% v/v normal saline. The dilution of other ions was the same as the 80% level (except that it was 90% for chloride). In this modified saline, concentrations of the added glucose and choline were cut in half (glucose: 121  mM/l, choline: 57  mM/l).

Normal *Aplysia* saline was used in the two outer chambers. The chambers were isolated using Vaseline Petroleum Jelly (Unilever, Connecticut. We incorporated a conductivity meter (${\mathrm{CON}}^{6+}$, OAKTON Instruments, Illinois) into the perfusion line to monitor the solution’s concentration based on the conductivity difference between the modified saline and normal *Aplysia* saline, which is caused by the dilution of ion concentrations. The solution in the middle chamber was considered correctly perfused when a stable conductivity reading equal to the reference value of the modified saline was achieved. All test solutions were kept at room temperature (∼22°C) during the experiment for consistent conductivity measurements.

#### 2.2.3 Laser

The IR light was generated by a single-mode laser diode (λ=1485  nm, QFBGLD-1480-500, QPhotonics, Michigan) driven by a diode driver and thermoelectric cooler controller (6340-4A, Arroyo Instruments, California). The laser was coupled into a 600-μm multimode optical fiber (P600-5 VIS-NIR, Ocean Insight, Florida), which was held in direct contact with the nerve by a micromanipulator. The 10-second laser pulse train (1250 Hz, 400  μs pulse width) was triggered by a DAQ device (USB-6218, National Instruments, Texas). Radiant exposure level was calculated using the average power measured by a power meter (PS19Q, Coherent, California).

### 2.3 Thermal Measurements

To measure the IR-induced temperature rise under different IR thresholds, we used a thermal camera (FLIR A325sc, Oregon) to measure the temperature distribution of an approximated midplane solution as previously reported.^4^^,^^5^ In brief, we cut a Petri dish along its midline and covered the cut end with a thin flat cover glass. The dish was filled with normal *Aplysia* saline or the modified saline to mimic the experimental conditions. A 600-μm optical fiber was positioned vertically aiming downward so that its en-face diameter was bisected by the glass–water interface and in contact with the cover glass’s upper edge. With calibration, the measured temperature distribution on the outer surface of the thin cover glass is an approximation to the actual midplane temperature distribution in the solution. When the laser light was applied through the fiber, we extracted the maximum temperature from the thermal distribution to represent the thermal condition that a nerve may experience during the IR application. The same IR parameters as applied in the inhibition experiments were used to determine temperature changes induced by IR inhibition alone versus IR inhibition combined with glucose.

### 2.4 Experimental Design and Analysis

We used separate groups of nerves to test the hypothesis that the IR inhibition threshold can be lowered by combining each type of isotonic ion-replaced saline. Using high-glucose saline as an example, here are the testing steps: (1) verify that high-glucose saline could not induce inhibition; (2) identify the IR threshold ($I_{\text{combined}}$) for inhibition when IR was applied along with the high-glucose saline in the middle chamber; (3) verify that the identified IR threshold ($I_{\text{combined}}$) in the previous test was a subthreshold IR level that alone could not induce inhibition; (4) identify the IR threshold ($I_{IR}$) in normal *Aplysia* saline. Control tests to generate CAPs in normal *Aplysia* saline before and after all the inhibition tests were conducted to assess the nerve’s health. More specifically, in each 105-second experimental trial, three 10-second applications of IR light were applied at the 10th, 45th, and 80th seconds of the trial. The IR power was carefully ramped up across trials to identify the IR threshold for inhibition.

We calculated and compared the rectified area under the curve (RAUC) of the CAPs to verify that the IR thresholds under different testing solutions correspond to the same inhibition level. Since heat accumulates during the first 6 seconds, only the CAPs from the 7th to 10th second of each IR application were analyzed. For the initial and final trials without IR application, CAPs at the identical time points were used, which helped take account of the very gradual rundown of the CAP due to electrical stimulation over time. To make a comparison of inhibition strength across nerve samples, the average RAUC of each nerve’s response at the designated time points was normalized to the average RAUC of the first 10 CAPs of its first normal saline control test.

For each group of nerves tested with given modified saline, we compared the IR thresholds in the modified saline versus in normal saline, once the normalized RAUC response confirmed the consistency of IR inhibition strength. A one-tailed t-test was conducted on the IR thresholds to see if it was lowered by the given modified saline. Finally, we calculated the percent change of IR threshold as follows:

$$\text{Percent Change of}\;\mathrm{IR}\;\text{threshold}=(\frac{I_{\text{combined}}}{I_{IR}}-1)\times 100\;(\%).$$ (1)

We conducted a repeated measures of analysis of variance (ANOVA) test on those percentage changes of IR threshold under different types of modified saline to explore if there was a preferable method. For the quantification and statistical analysis of size selectivity, we used the same method as previously described.^5^ The CAPs were divided into fast-conducting and slow-conducting regions at a point of low variability. Next, we calculated the normalized RAUC within each region. For both fast and slow regions, we categorized a region as inhibited during a given CAP only when the normalized RAUC was lower than 0.6. Twenty-eight CAPs (four per animal) were analyzed for each type of modified saline. The chi-squared test was used to compare the inhibition effect on fast-conducting large-diameter axons and slow-conducting small-diameter axons.

## 3 Results

**Fig. 2:**
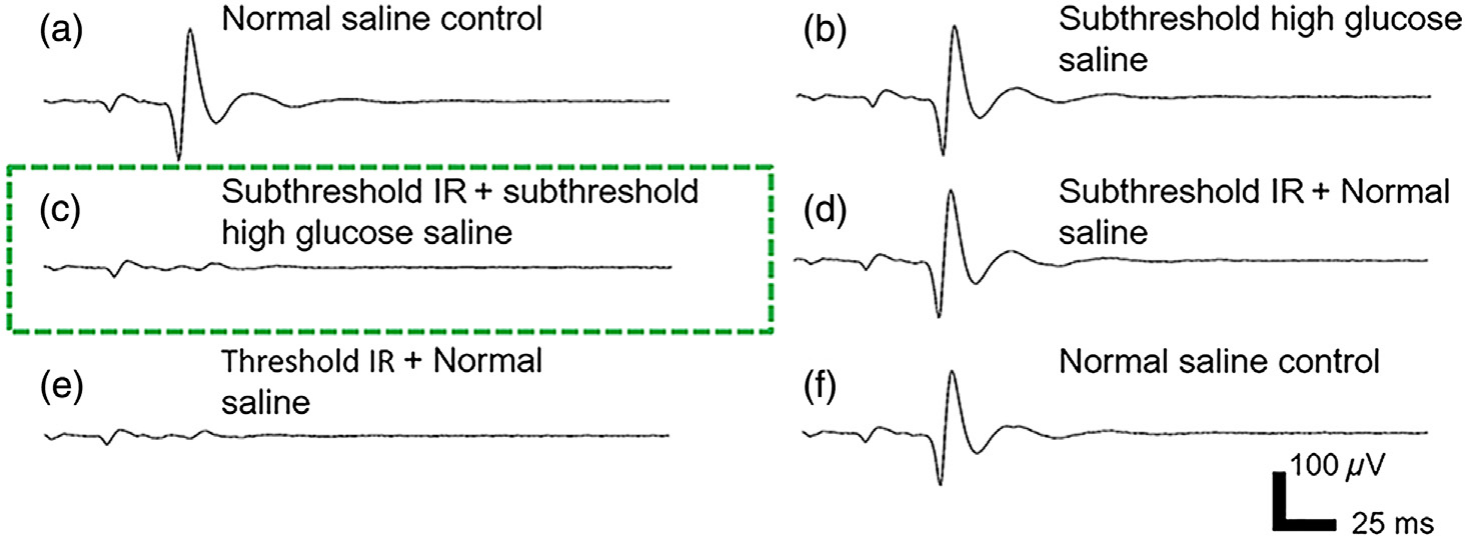
Combined inhibition. A typical raw data set showing that subthreshold IR combined with subthreshold high-glucose saline can achieve full inhibition similar to the threshold level IR alone in normal saline. (a) A normal saline control experiment elicited a full CAP from electrical stimulation. (b) Subthreshold high-glucose saline did not elicit a significant inhibitory effect. (c) When subthreshold IR was combined with subthreshold high-glucose saline, an almost full inhibitory effect was observed. (d) When the same subthreshold IR level was applied alone, it did not show a significant inhibitory effect. (e) When IR was applied in normal saline alone, a higher power level was needed to induce an inhibition effect similar to (c). (f) A normal saline control test after all the tests showed a CAP similar to (a), suggesting the nerve’s health was not affected.

**Fig. 3:**
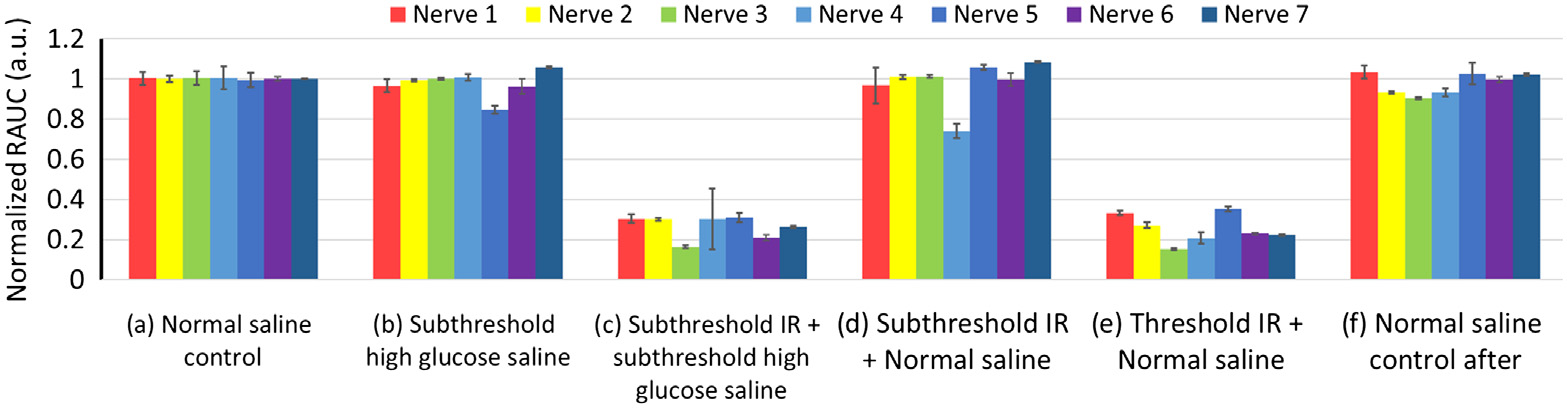
Normalized RAUC results from seven nerves. For each nerve, the absolute RAUC number was normalized to the averaged RAUC from the first 10 CAPs of the initial normal saline control experiment. Groups (b) and (d) show little to no inhibition with a RAUC well above 0.6, suggesting that both subthreshold high-glucose saline and subthreshold IR cannot inhibit the action potential conduction alone. Both groups (c) and (e) show a strong inhibitory effect (normalized RAUC<0.6) suggesting that the combined modality (c) can achieve a similar inhibition as IR alone (e) but requiring lower IR levels. The nerve’s health was not affected by the different treatments as groups (a) and (f) did not show significant differences.

All three types of subthreshold ion-replaced saline were able to lower the IR inhibition threshold. For example, Fig. 2 shows a typical dataset in which the IR inhibition threshold was lowered by high-glucose saline. As positive controls, neither subthreshold high-glucose saline nor subthreshold IR radiant exposure ($12.2\;\mathrm{mJ}/{\mathrm{cm}}^{2}$) alone could inhibit the CAPs [Figs. 2(b) and 2(d)]. In contrast, when the same subthreshold IR radiant exposure was combined with the subthreshold high-glucose saline, full inhibition was achieved [Fig. 2(c)]. The radiant exposure threshold for IR inhibition alone in normal *Aplysia* saline was higher [$13.7\;\mathrm{mJ}/{\mathrm{cm}}^{2}$, Fig. 2(e)]. The CAPs in the normal saline control tests before and after inhibition tests [Figs. 2(a) and 2(f)] did not show an obvious difference. The experiments were repeated seven times for high-glucose saline and the normalized RAUC results are shown in Fig. 3.

The RAUC result confirmed that IR combined with high-glucose saline achieved the same inhibitory effect as IR alone, but required lower IR levels. A paired t-test between the combined inhibition and IR inhibition [Figs. 3(c) and 3(e)] did not show a significant difference (p=0.539). Neither subthreshold modality lowers the RAUC below 0.6, a level we considered as obvious inhibition [Figs. 3(b) and 3(d)]. The RAUC for the two control tests [Figs. 3(a) and 3(f)] did not show a significant difference in the paired t-test (p=0.274). Thus, inhibition tests did not significantly affect the nerve’s health. The same RAUC analysis was conducted on the results from the high-choline saline group and high-glucose/high-choline saline group, and our paired t-tests showed consistent inhibition between groups c and e for each type of modified saline. This enabled accurate measurements of the reduction in IR threshold or temperature.

**Fig. 4:**
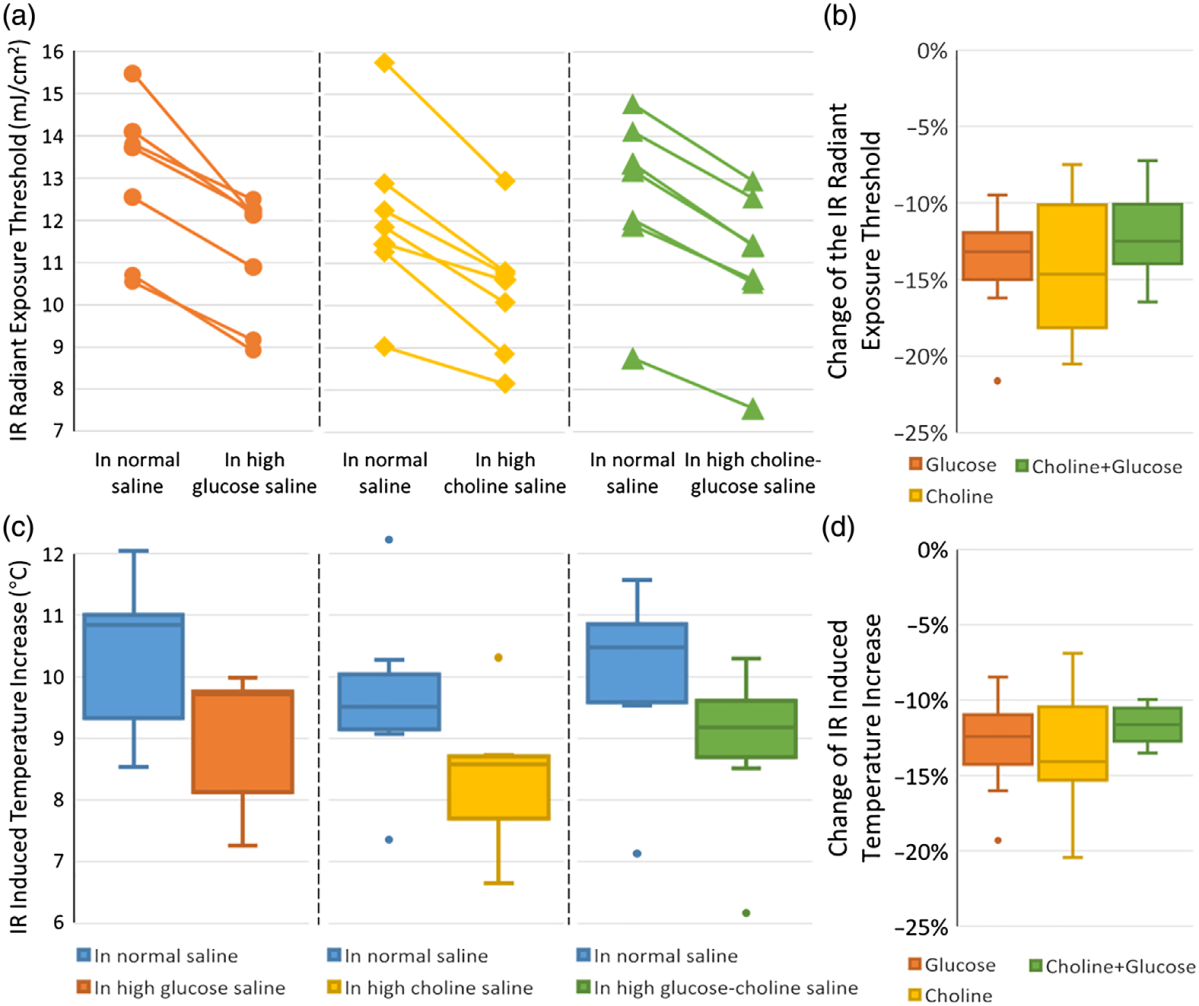
The IR radiant exposure threshold and IR induced maximum temperature change in normal *Aplysia* saline versus in each type of isotonic ion-replaced saline. (a) The IR radiant exposure threshold for inhibition in each type of isotonic ion-replaced saline showed a significant drop compared with the threshold in normal *Aplysia* saline. Each line indicates a pair of IR threshold tested on the same nerve in different conditions. (b) The changes in IR thresholds were not significantly different between different types of modified saline. (c) The box-whisker plot of maximum IR-induced temperature rise showed a significant drop when IR is applied in each type of modified saline compared with the reading in normal *Aplysia* saline. (d) The changes in IR-induced maximum temperature increase were not significantly different between different types of modified saline.

Infrared radiant exposure thresholds for inhibition were significantly lower (one-tailed t-test, p<0.05) in all three types of ion-replaced saline than in normal *Aplysia* saline [Fig. 4(a)]. The percentage changes of IR threshold were: 14.1±3.7% for high-glucose saline, 14.3±4.5% for high-choline saline, 12.6±1.3% for high-glucose/high-choline saline [Fig. 4(b)]. A one-way ANOVA test of the IR threshold changes across different types of modified saline did not show a significant difference (p=0.724). The thermal camera measurement showed a significant drop (one-tailed t-test, p<0.05) of the IR-induced maximum temperature rise in all three types of modified saline compared to the temperature needed for IR alone in normal *Aplysia* saline [Fig. 4(c)]. The percentage changes of the IR induced maximum temperature rise were: 12.9±3.4% for high-glucose saline, 13.3±4.1% for high-choline saline, 11.7±1.3% for high-glucose/high-choline saline [Fig. 4(d)]. A one-way ANOVA test of the temperature threshold changes across different types of modified saline did not show a significant difference (p=0.653).

**Fig. 5:**
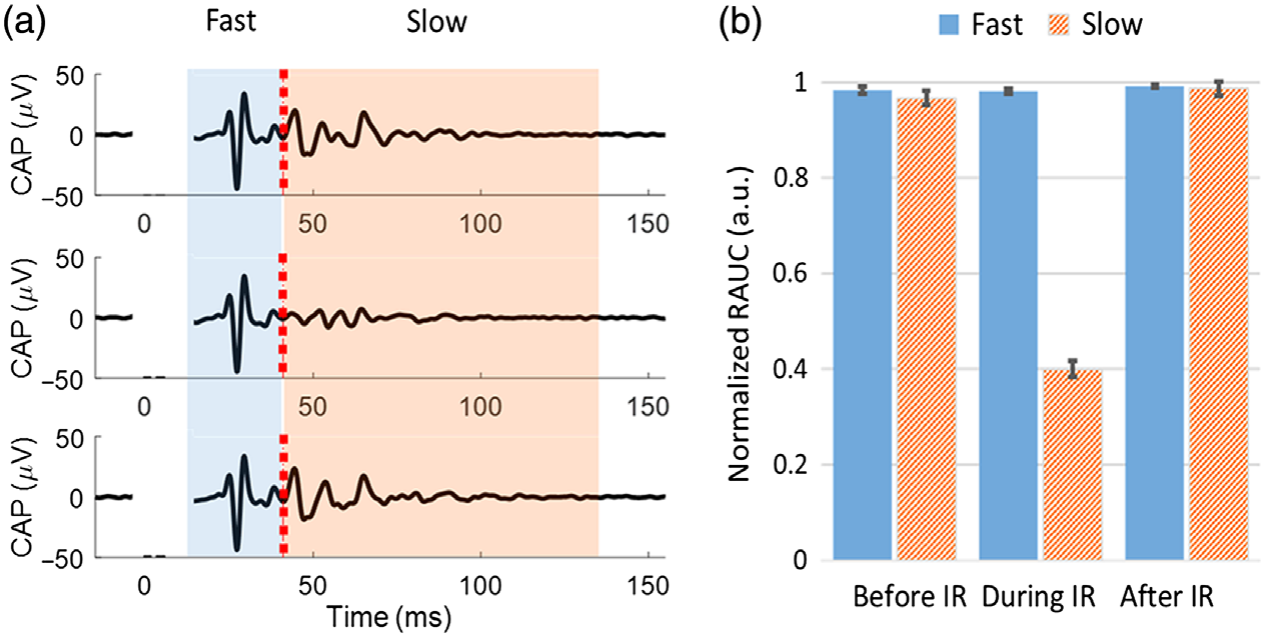
Selective inhibition on slow-conducting small-diameter axons when IR was applied in subthreshold high-glucose/high-choline saline. (a) Partial block of the CAP components carried by the slower-conducting small-diameter axons. The red dashed line indicates the low variability point for separation. The artifact caused by electrical stimulation was blanked. (b) The normalized RAUC quantification in (b) showed that only the slow components were inhibited by the IR application.

Selective inhibition of slow-conducting small-diameter axons was observed with each type of isotonic ion-replaced saline. A typical CAP is shown in Fig. 5(a) for IR combined with high-glucose/high-choline saline. As shown in Fig. 5(b), the fast-conducting components carried by the large-diameter axons were not inhibited while the slow-conducting components carried by the small-diameter axons were inhibited (two-sample t-test, p<0.05). For all three types of modified saline, we conducted the same RAUC calculation and categorization of the fast/slow components of the CAPs during IR application. After the categorization, the cumulative number of CAPs in response to the IR application combined with each type of modified saline is shown in Table 1.

**Table 1:** Cumulative number of categorized CAPs when IR was applied in combination with different types of isotonic ion-replaced saline.

Type of modified Saline	Type of response	Number of CAPs	
		Fast-conducting, large-diameter	Slow-conducting, small diameter
High-glucose saline	Uninhibited	18	4
	Inhibited	10	24
High-choline saline	Uninhibited	24	0
	Inhibited	4	28
High-glucose/high-choline saline	Uninhibited	24	8
	Inhibited	4	20

Using chi-squared tests, all three types of modified saline showed statistically significant inhibition preference on the slow-conducting regions as compared to the fast-conducting regions (p<0.05 for all three groups). This suggests that the size-selectivity of IR inhibition was preserved when combined with isotonic ion-replaced saline, which is similar to our previous results using IR alone.^5^

## 4 Conclusion and Discussion

The results confirmed our hypothesis that combining IR inhibition with isotonic ion-replacement by glucose and/or choline can lower the required radiant exposure threshold for IR inhibition. The combined method can lower the IR-induced temperature increase, which can potentially reduce the risk of damage due to acute IR-induced heating. More importantly, the size selectivity on small-diameter axons of IR inhibition was preserved when combined with isotonic ion replacement. The combined method appears to be safe in the acute setting since the inhibition effect is reversible after each inhibition modality.

For all three types of modified saline, we observed a similar reduction effect on the IR threshold, suggesting that the similar level of dilution to the ions in the modified saline was the major reason for the reduction effect. These results are consistent with our previous mathematical analysis^5^ that any modality (e.g., isotonic ion replacement used in the combination here) that primarily works on the membrane will selectivity affect small-diameter axons. It is important to point out that when glucose and choline were both applied in half the osmotic concentrations that they had been applied alone, the modified saline still induced a similar reduction in IR threshold as the reduction when glucose or choline was applied alone. This finding provides a basis for future studies using several solutes for isotonic ion replacement to lower the IR inhibition threshold, keeping each solute within safe concentrations. It has not escaped our notice that diabetes patients already have an elevated glucose level that is 2 to 4 times higher than normal, which can make neural conduction more vulnerable and cause chronic neuropathy or functional toxicity.^26^^–^^28^ An administration of choline and/or other ion-substitution solutes, in addition to the already elevated glucose level, may lower the threshold for IR inhibition or other heating modalities to treat the neuropathic pain caused by diabetes.

The present work is an acute study of IR inhibition combined with isotonic ion replacement. To accurately find the IR threshold for each condition, six tests were performed (see Fig. 3), which took multiple rounds of IR application and fluid changing. This experimental design allowed us to measure two IR inhibition thresholds for each nerve: one in the normal saline and one in the test solution while ensuring that the nerve’s health remained consistent (e.g., control tests did not show significant differences in RAUC) throughout the experimental process. The results confirmed that the selected substrate concentrations, although higher than therapeutic levels,^29^ were safe for the present acute *ex vivo* tests. More importantly, those acute tests demonstrated that isotonic ion replacement can lower the IR threshold regardless of the substrate or substrate mix used for the ion replacement. This finding will serve as the basis for further chronic studies of IR inhibition combined with isotonic ion replacement to explore: (1) the stability of the isotonic ion-replaced saline’s reduction effect on the IR inhibition threshold over the repeated test; (2) multiple ion-replacement compounds, in combination, could be used at concentrations that were suitable for potential translational applications; (3) the ratio between the inhibition threshold and the damage threshold in normal saline versus in isotonic ion-replaced saline.

Infrared neuromodulation has been combined with other modalities to lower the IR threshold. For example, Duke et al.^15^ demonstrated that combining electrical and IR neural stimulation (INS) could reduce the threshold for INS. Our data highlight the potential practical benefit of combining IR inhibition with other inhibitory modalities. The combination of IR inhibition with isotonic ion replacement lowered the IR threshold and the related temperature rise while preserving the size selectivity on the small-diameter axons. These results will guide the development of a more effective size-selective IR inhibition modality for future research and translational applications.
